# Ontogenetic Profile of the Expression of Thyroid Hormone Receptors in Rat and Human Corpora Cavernosa of the Penis

**DOI:** 10.1111/j.1743-6109.2009.01701.x

**Published:** 2010-04

**Authors:** Eleonora Carosa, Stefania Di Sante, Simona Rossi, Alessandra Castri, Fabio D'Adamo, Giovanni Luca Gravina, Piero Ronchi, Zdenek Kostrouch, Susanna Dolci, Andrea Lenzi, Emmanuele A Jannini

**Affiliations:** * Course of Endocrinology and Medical Sexology, Department of Experimental Medicine, University of L'AquilaL'Aquila, Italy; † Urology Functional Unit, “Villa Igea” ClinicAncona, Italy; ‡ Laboratory of Molecular Pathology, Institute of Inherited Metabolic Disorders, Charles UniversityPrague, Czech Republic; § Chair of Anatomy, Department of Public Health, II University of Rome “Tor Vergata”Italy; ¶ Chair of Endocrinology, Department of Medical Pathophysiology, 1^st^ University of Rome “La Sapienza”Italy

**Keywords:** Corpora Cavernosa, Thyroid Hormone Receptor, Erectile Dysfunction, Thyroid Disorders and Sexual Dysfunction

## Abstract

**Introduction:**

In the last few years, various studies have underlined a correlation between thyroid function and male sexual function, hypothesizing a direct action of thyroid hormones on the penis.

**Aim:**

To study the spatiotemporal distribution of mRNA for the thyroid hormone nuclear receptors (TR) α1, α2 and β in the penis and smooth muscle cells (SMCs) of the corpora cavernosa of rats and humans during development.

**Methods:**

We used several molecular biology techniques to study the TR expression in whole tissues or primary cultures from human and rodent penile tissues of different ages.

**Main Outcome Measure:**

We measured our data by semi-quantitative reverse transcription polymerase chain reaction (RT-PCR) amplification, Northern blot and immunohistochemistry.

**Results:**

We found that TRα1 and TRα2 are both expressed in the penis and in SMCs during ontogenesis without development-dependent changes. However, in the rodent model, TRβ shows an increase from 3 to 6 days post natum (dpn) to 20 dpn, remaining high in adulthood. The same expression profile was observed in humans. While the expression of TRβ is strictly regulated by development, TRα1 is the principal isoform present in corpora cavernosa, suggesting its importance in SMC function. These results have been confirmed by immunohistochemistry localization in SMCs and endothelial cells of the corpora cavernosa.

**Conclusions:**

The presence of TRs in the penis provides the biological basis for the direct action of thyroid hormones on this organ. Given this evidence, physicians would be advised to investigate sexual function in men with thyroid disorders. **Carosa E, Di Sante S, Rossi S, Castri A, D'Adamo F, Gravina GL, Ronchi P, Kostrouch Z, Dolci S, Lenzi A, and Jannini EA. Ontogenetic profile of the expression of thyroid hormone receptors in rat and human corpora cavernosa of the penis. J Sex Med 2010;7:1381–1390.**

## Introduction

Thyroid hormone receptors (TR_s_) are part of the nuclear receptor superfamily. TR_α_ and TR_β_ are the products of two distinct genes that are further differentially spliced into TR_α1_, TR_α2_[1,2], TR_β1_, and TR_β2_[3,4]. TR_α1_ and TR_β1_ are widely expressed and act as thyroid hormone-dependent transcription factors, inducing or repressing gene expression in response to triiodothyronine (T_3_). The differentially spliced product TR_α2_ does not bind the hormone and exerts a dominant negative effect on the action of other TR_s_[5,6]. The TR α and β forms are expressed in a distinct but often overlapping pattern, suggesting that they may mediate both individual and common functions. Several specific functions of single TR isoforms have been identified using knockout mice for TR_α_ and/or TR_β_. In fact, the importance of TR_α1_ in post-natal development and cardiovascular functions is well known [7–10], whereas TR_β2_ is fundamental in the regulation of pituitary–thyroid axis development [11,12]. The influence of thyroid hormones on mating behavior has also been investigated in knockout mice. TR_α1_^−/−^ animals showed hyposexual behavior, while TR_β_^−/−^ mice showed significantly enhanced sexual behavior [13].

Male reproductive and sexual function appears to be controlled by thyroid hormones in both animals and humans [9,14]. Although this area has been neglected in the past, various studies over the past few years have underlined a correlation between thyroid function and male sexual function. Premature ejaculation and erectile dysfunction (ED) are frequent findings in thyroid disease [15–19], suggesting the direct involvement of thyroid hormones in the physiology of male sexual function. However, despite the now well-established association between thyroid function and male sexual activity, there is a dearth of research exploring the locoregional expression of TR_s_ in the penis. For this reason, we studied the expression pattern of the individual isoforms of TR_s_ in rat and human penis during the development.

## Materials and Methods

### Animals

Male Wistar rats were reared in our institute's animal facilities, and all experimental protocols were approved by the local ethics committee. The penises from rats of different ages [3–6, 20, 60, and 300 days *post natum* (d*pn*)] were excised and separated from the cutis and preputial glands and used for cell cultures, for RNA extraction (stored in liquid N_2_), or fixed for immunohistochemistry. We used, in separate experiments, sexually immature (under 30 days old) and adult (until 300 days old) male rats [20]; we also focused our attention on peripubertal rats (20 days old), where androgen receptor is expressed in corporal cavernosa SMCs [21].

### Patients

After protocol approval by the local Clinical Investigation Committee, human corpora cavernosa were obtained from three impotent men (age 71 ± 10.4) at the time of penile prosthesis implantation. After surgery, the corpora cavernosa were immediately used for cell culture preparation or frozen in liquid N_2_ for RNA extraction [22].

### Immunohistochemistry

Penises from 60-day-old animals were fixed in 4% paraformaldehyde in PBS (140 mM NaCl, 50 mM phosphate buffer pH 7.2) and embedded in paraffin. Immunostaining was carried out on 4-µm thick sections of 60-day-old rat penis. Sections were deparaffinized in xylene and rehydrated (in isopropylalcohol and ethanol–water). Next, sections were boiled in Target Retrieval Solution High pH (Dako Cytomation, Carpinteria, CA, USA) for 40 minutes at 95°C in a water bath, and then the sections were left for 20 minutes on bench for temperature equilibration. Next, the sections were placed in deionized water. The endogenous peroxidase was blocked in the water solution of hydrogen peroxide (3%) containing 0.1% sodium azide for 30′ and again transferred to water. The TR_α1_ monoclonal antibody was a kind gift from Dr. Onno Bakker [23]. The primary antibody was diluted in Dako Real, Antibody Diluent at 1:100 dilution. The sections were incubated with the antibody overnight at 4°C. After washing in TBS (50 mM Tris, 150 mM NaCl, pH 7.6) (three times for 3 minutes each), the sections were incubated with a secondary antibody for 30 minutes at room temperature (using the EnVision System HRP from Dako). Finally, the sections were washed again in TBS (three times for 3 minutes), and the antibody was visualized using the DAB Dako Liquid DAB + Substrate Chromogen System (Dako) (5 minutes incubation). Sections were washed shortly afterwards with water. In some cases, nuclei were controstained using Harris Hematoxylin. Sections were dehydrated in alcohol and xylen, then mounted in Solacryl (Penta, Prague, Czech Republic). Slides were observed in Olympus BX60 microscope (Center Valley, PA, USA) equipped with the DP30 CCD camera (Olympus, Center Valley, PA, USA).

### Corpus Cavernosum Smooth Muscle Cell Cultures

Primary cultures of rat *corpus cavernosum* smooth muscle cells were obtained by modifying the method used by Krall et al. [24]. Briefly, small pieces of corpora cavernosa (about 1 mm square) were cut from penises excised from rats of different ages and/or from human corpora cavernosa. Sterile forceps were used to press the fragments onto the bottom of cell culture wells containing Dulbecco's Modified Essential Medium (D-MEM) (Gibco/Invitrogen, Carlsbad, CA, USA) + 20% Fetal Bovine Serum (FBS; Sigma, St. Louis, MO, USA), 2 mmol/L glutamine (Gibco), 100 IU/mL penicillin, and 100 µg/mL Streptomycin (Gibco). The fragments were incubated undisturbed for 4–6 days at 37°C in fully humidified atmosphere with 5% CO_2_. After 4–6 days, the culture medium was replaced with fresh medium. The cultures were incubated undisturbed until 50% confluence was reached, when the remaining tissue fragments were removed and the medium changed. When the cultures were confluent, the cells were split 1:3 and plated in D-MEM + 10% FBS. This transfer procedure was repeated for subsequent passages. With this method, we obtained a culture of smooth muscle cells from 3 (rCC3) and 20 (rCC20) d*pn* rat corpora cavernosa and from human corpora cavernosa (hAdult CC). We characterized rat primary cultures by immunostaining with an antibody anti-α-smooth muscle actin and with an antibody against the endothelial-specific marker CD31, as previously described in Carosa et al. [21]. Endothelial cell contamination was less than 10% [21]. We also characterized the human culture with immunostaining with anti-α-smooth muscle actin antibody (data not shown). These cells were used within the 10th passage.

### RNA Extraction and Northern Blot

Total RNA was prepared by homogenization from rat penis, human corpora cavernosa and corpus cavernosum cells from different aged rats or humans and extracted with RNeasy Kit with on-column deoxyribonuclease (DNase) digestion (Qiagen, Milan, Italy). RNA purity and integrity was checked spectroscopically and by gel electrophoresis. Human fetal penile smooth muscle cells (hfPSMC) were kindly provided by Prof. Maggi (University of Florence, Italy). hfPSMC were cultured as described in Granchi et al. [25] and used for the RNA preparation.

For Northern blot analysis, RNA samples (20 µg) were denatured, separated on 1% formaldehyde–agarose gel, transferred on nylon membranes (Amersham—GE Healthcare Technologies, Milan, Italy), and probed at high stringency with the [32] P-labeled rat TR cDNAs. Specifically, a 442 bp fragment obtained from PCR amplification of TR_β_ plasmid [26] using TR_β_ up (5′-CAA TCA CCA GAG TGG TGG ATT TCG CCA-3′) as the upstream primer and TR_β_ 1574do (5′-ATC CGC AGA TCT GTC ACC TT-3′) as the downstream primer was used to probe the presence of the TRβ. The filter was then hybridized with the full-length cDNA fragment for TR_α_[26]. Normalization was obtained by hybridizing the filter with a glyceraldehyde-3-phosphate dehydrogenase (GAPDH) probe. The probes used for hybridization of human RNA are described in Jannini et al. [27].

Hybridization was carried out in QuikHyb (Stratagene, La Jolla, CA, USA) as recommended by the manufacturer. Autoradiograms were analyzed densitometrically, and the results were expressed as arbitrary units of optical densities. The densitometric analysis was performed using the ImageJ 1.25s program (NIH, Bethesda, MD, USA; http://rsb.info.nih.gov/ij/).

### Semiquantitative RT-PCR Analysis

First strand complementary DNA was produced using 2 µg of total RNA for each sample, in the presence of Superscript II reverse transcriptase (Invitrogen, Carlsbad, CA, USA) and poly-d(T)_12-18_ primer (Invitrogen). The cDNA thus obtained was used as template for PCR amplification of the TR_s_ (Table 1) end GAPDH (GAPDHup 5′-TGA AGG TCG GTG TGA ACG GAT TTG GC-3′ and GAPDHdo 5′-CAT GTA GGC CAT GAG GTC CAC CAC-3′). GAPDH was used as an internal control to analyze RNA integrity and quantity. The PCR was performed in the presence of Go-Taq DNA polymerase (Promega, Madison, WI, USA; 2.5 units per reaction) in the Px 2 thermal cycler (Thermo Electron Corporation, Basingstoke, UK) for 30 cycles at 94°C for 1 minute, 60°C for 1 minute, and 72°C for 1 minute for TRs amplification, and 20 cycles at 94°C for 45 seconds, 55°C for 45 seconds and 72°C for 45 seconds for GAPDH amplification. Genomic contamination was excluded by running the PCR without RT and drawing the primers in different exons. The amplified fragments were separated in 1% agarose gel and acquired using the microDOC Gel Documentation System (Cleaver Scientific Ltd, Rugby, Warwickshire, UK). The densitometric analysis was performed using the ImageJ 1.25s program (NIH).

**Table 1:** Oligo sequences for TRs amplification

Gene name	Accession number	Oligo name	Sequence	Fragment length (bp)
TR_α1_	NM_199334 for human and NM_001017960 for rat	TR_α_ common	5′-GCG TAA GCT GAT TGA GCA GA-3′	758
		TR_α1_do	5′-CCT CAA AGA CCT CGA GGA AG-3	
TR_α2_	NM_003250for human and NM_031134 for rat	TR_α_ common	5′-GCG TAA GCT GAT TGA GCA GA-3′	824
		TR_α2_do	5′-GAA CAA CAT GCA TTC CGA GA-3′	
TR_β_	NM_000461for human and NM_012672 for rat	TR_β_936up	5′-GGA ATG GGA GCT CAT CAA AAC-3′	639
		TRβ1574	5′-ATC CGC AGA TCT GTC ACC TT-3′	

### Statistical Analysis

Continuous variables were presented as a mean and standard deviation (SD) and analyzed using Student's *t*-test for unpaired data. All statistical tests were two-tailed. A *P* value of <0.05 was considered as statistically significant. The results represent a mean of at least three separate experiments.

## Results

**Figure 1:**
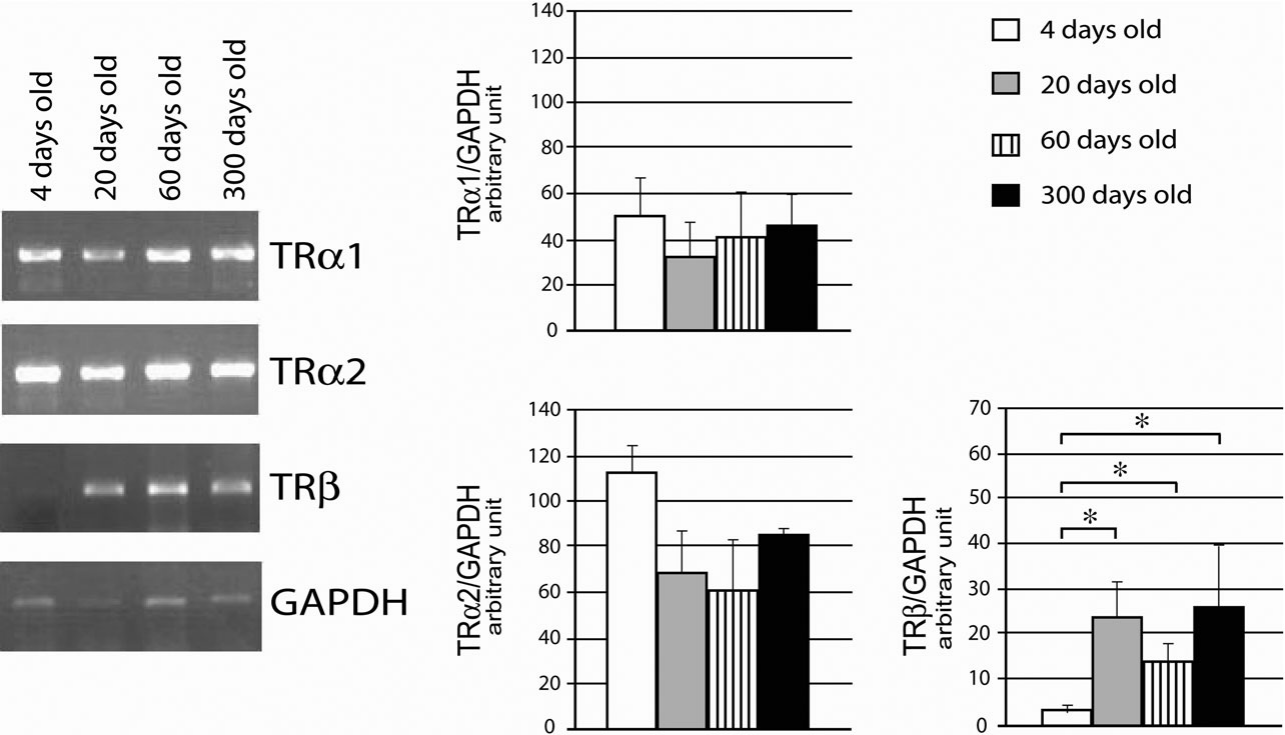
RT–PCR analysis of TR_α1_, TR_α2_, and TR_β_ expression in penises from rats of different ages. The PCR products are derived from total RNA from rat penises of 4, 20, 60, and 300 dpn rats using specific primers for TR_s_ and GAPDH. GAPDH mRNA amplification was performed to verify the integrity of the extracted total RNA. Densitometric evaluation of TR_s_ mRNA expression over the GAPDH housekeeping gene was obtained from band intensity of RT-PCR products. The densitometric analysis of TR_α1_, TR_α2_, and TR_β_ from three independent experiments ± SD (*P < 0.05) is shown.

Thyroid hormone receptor expression and development-related regulation was evaluated in rat corpora cavernosa by semiquantitative RT-PCR analysis of the penises of rats of different ages. Total RNA extracted from the penises of 4-, 20-, 60-, and 300-day-old rats was amplified using specific primers for TR_α1_, TR_α2_, and TR_β1_. As shown in Figure 1, the two products of differential splicing TR_α1_ and TR_α2_ were both expressed without any significant age-related difference. In contrast, the expression of TR_β1_ increased 6.6 times (3.62 ± 1.15 vs. 23.92 ± 7.81 a.u.; *P* = 0.043) from perinatal rat penis to puberty, remaining high during adulthood (Figure 1). The level of TR_α2_ was higher than TR_α1_ in all ages studied, with a constant α_2_/α_1_ ratio of 1.88 ± 0.34, demonstrating no changes in the differential splicing machinery.

**Figure 2:**
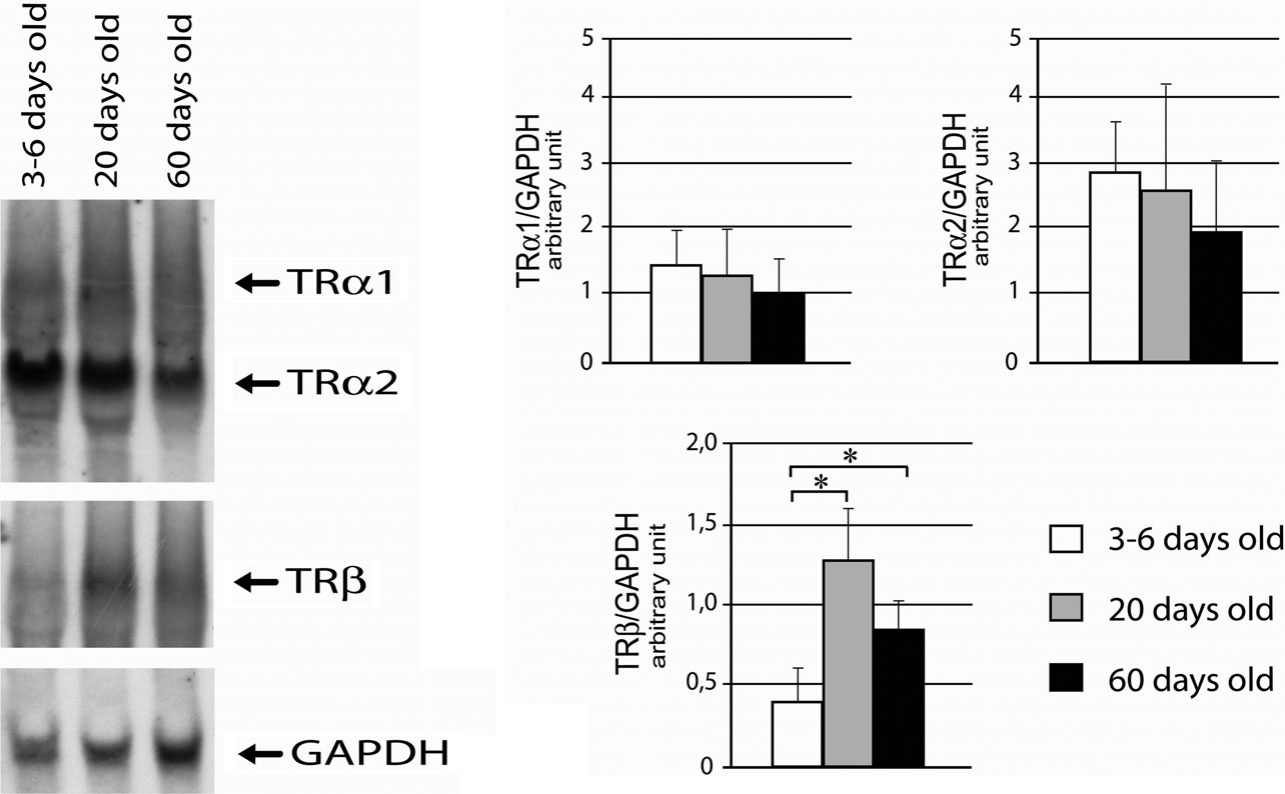
Expression of mRNA from various TR isoforms in rat penis during development and adulthood. Representative Northern blot analysis of 20 µg of total RNA extracted from rat penises of 3–6, 20, and 60 dpn animals. Samples were separated in denaturing gel, blotted on nylon filter, and hybridized with the cDNA fragments corresponding to the indicated TR_s_ or GAPDH. Densitometric evaluation of TR_s_ mRNA expression over the GAPDH housekeeping gene was obtained from band intensity. The densitometric analysis of TR_α1_, TR_α2_, and TR_β_ from three independent experiments ± SD (*P < 0.05) is shown.

To analyze the expression pattern of all TR isoforms in more detail, Northern blot experiments were performed on total RNA extracted from whole penises. The full-length cDNA fragment of TR_α1_, containing the common sequence of TR_α1_ and TR_α2_, was used to probe total RNA from the penises of 3–6-, 20-, and 60-day-old rats. The 5.5 kb band signal corresponding to α_1_ and the 2.8 kb band corresponding to α_2_ were both present in all samples (Figure 2), with no significant age-related difference. The levels of TR_α2_ were higher than of TR_α1_ in all ages studied and the ratio between them was similar to that found with RT-PCR (1.96 ± 0.02). This is in the agreement with the absence of development-related regulation. The same membranes were rehybridized with a PCR fragment containing 442 bp of TR_β1_ (Figure 2). A 6.0 kb band corresponding to TR_β1_ showed a sharp increase from perinatal to peripubertal age: a very faint band was seen in 3–6 day old rats, increasing threefold in rats of 20 d*pn* (0.41 ± 0.20 vs. 1.27 ± 0.32 a.u.; *P* = 0.024). The levels of TR_β1_ remained high in the penises of adult, 60-day-old, rats (0.85 ± 0.17 a.u.), further confirming the RT-PCR data. Hybridization to an GAPDH cDNA probe was used for normalization in order to estimate the relative amount of each TR isoform present at different ages.

**Figure 3:**
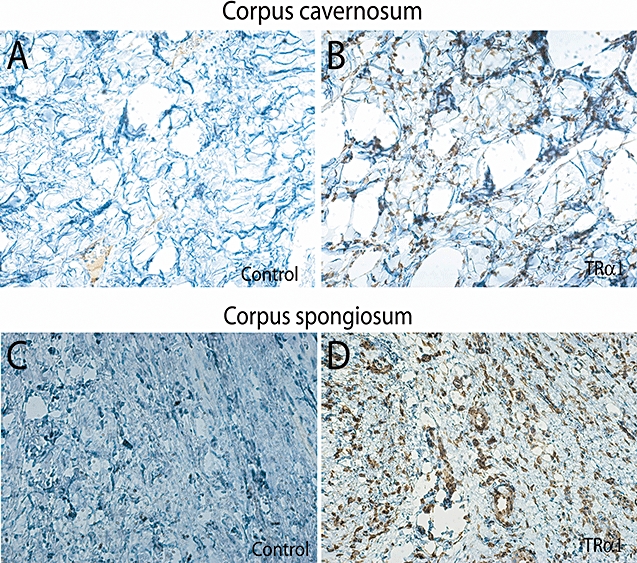
Localization of TR_α1_ obtained by immunohistochemistry experiment of histological sections of adult (60 dpn) rat penis. A and C represent the negative control. B and D are stained with the anti-TR_α1_ antibody (original magnification: 20×).

For TR localization, we performed immunohistochemistry experiments using an anti-TR_α1_ antibody. Widespread staining for this TR isoform was present in both endothelial and muscular cells of the corpora cavernosa (Figure 3B). A similar expression pattern was found in the corpus spongiosum, with TR_α1_ abundantly expressed in both muscular muscle and endothelial cells (Figure 3D).

**Figure 4:**
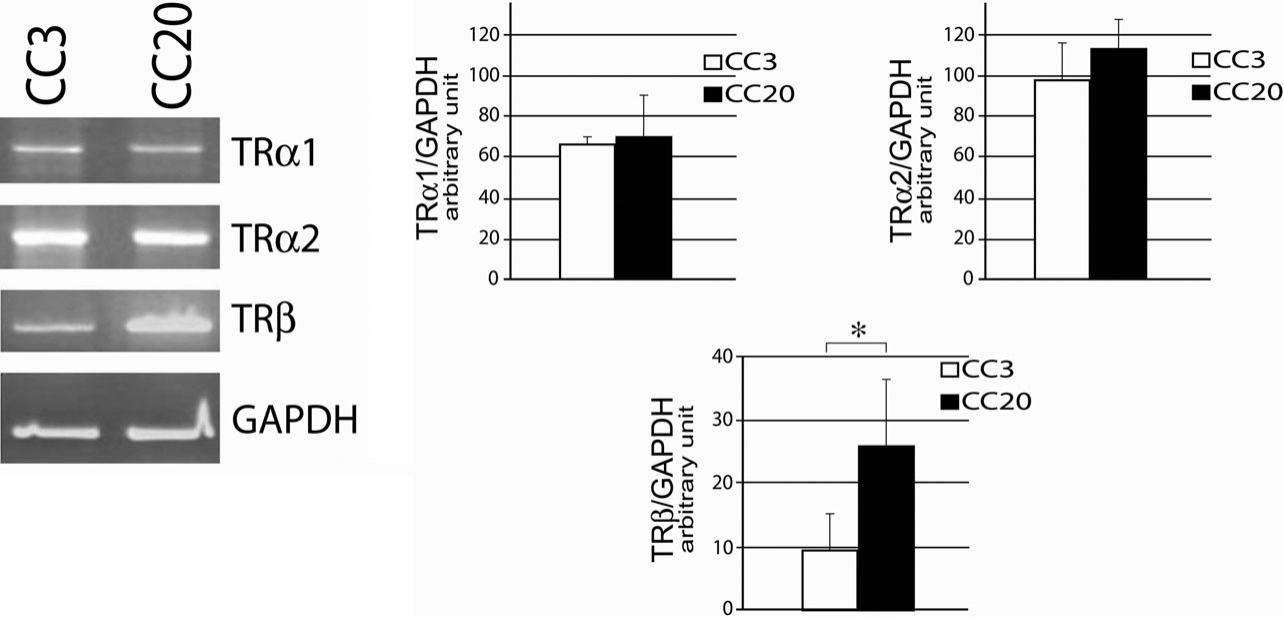
RT–PCR analysis of TR_α1_, TR_α2_, and TR_β_ expression in corpus cavernosum SMCs from rats of different ages. The products are derived from total RNA from rat corpus cavernosum SMCs from 3 (CC3) and 20 (CC20) dpn rats using specific primers for various TR_s_ and GAPDH. GAPDH mRNA amplification was performed to verify the integrity of the extracted total RNA. Densitometric evaluation of TR_s_ mRNA expression over the GAPDH housekeeping gene was obtained from band intensity of RT-PCR products. The histograms are calculated after normalization on GAPDH and on the basis of the amount of volume of PCR amplification loaded in the gel (1/5 of volume for TR_α1_ and TR_α2_, and 4/5 for the TR_β_ isoform). The densitometric analysis of TR_α1_, TR_α2_, and TR_β_ from three independent experiments ± SD (*P < 0.05) is shown.

As ED in thyroid diseases has been postulated to be due to a direct effect of thyroid hormones on SMCs [17,18], we focused our attention on these cells. We used SMCs obtained from the penises of 3- (rCC3) and 20-day-old (rCC20) rats (Figure 4), evaluating the presence of TR_α1_ and TR_α2_ mRNAs by RT-PCR. TR_α1_ and TR_α2_ levels remained unchanged, as did the α_2_:α_1_ ratio, which was almost the same as that observed for the whole penis (1.51 ± 0.09). As expected, a low level of TR_β1_ was found in SMCs from the corpora cavernosa of perinatal rats, increasing 2.7-fold in peripubertal rats, showing the same expression pattern as seen in penile tissues (9.45 ± 5.81 vs. 25.94 ± 10.71 a.u.; *P* = 0.046) (Figure 4).

**Figure 5:**
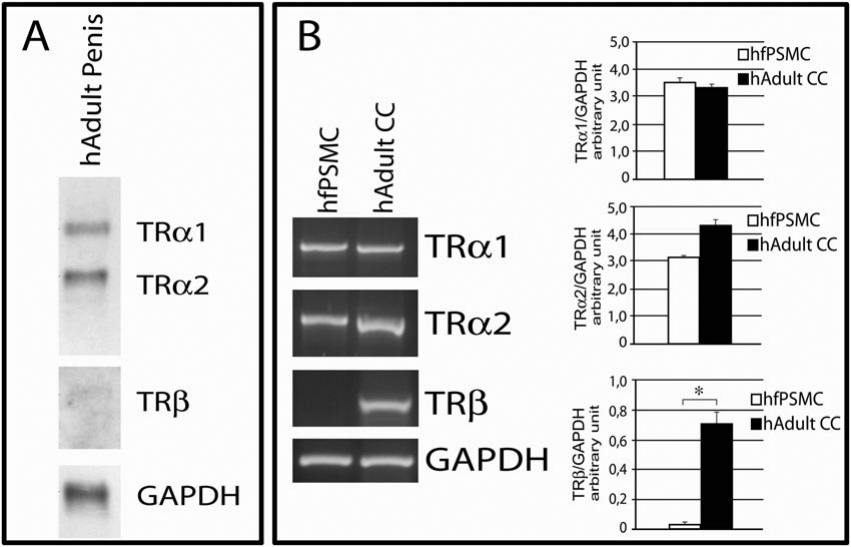
(A) Expression of mRNA from various TR isoforms in adult human corpora cavernosa. Representative Northern blot analysis of 20 µg of total RNA extracted from the penis of a 65-year-old man. Samples were separated in denaturing gel, blotted on nylon filter, and hybridized with the cDNA fragments corresponding to the indicated TRs or GAPDH probe to assess their integrity and concentration. (B) RT–PCR analysis of TR_α1_, TR_α2_, and TR_β_ expression in human corpus cavernosum SMCs. The products are derived from total RNA from human fetal (hfPSMC) and adult (hAdutCC) corpus cavernosum SMCs using specific primers for various TR_s_ and GAPDH. GAPDH mRNA amplification was performed to verify the integrity of the extracted total RNA. Densitometric evaluation of TR_s_ mRNA expression over the GAPDH housekeeping gene was obtained from band intensity of RT-PCR products. The histograms are calculated on the basis of the amount of volume of PCR amplification loaded in the gel (1/5 of volume for TR_α1_ and TR_α2_, and 4/5 for the TR_β_ isoform). The densitometric analysis of TR_α1_, TR_α2_, and TR_β_ from three independent experiments ± SD (*P < 0.05) is shown.

The expression of all TR_s_ was also studied in human corpora cavernosa by Northern blot (Figure 5A), confirming the presence of all these forms and isoforms in the adult penis. RT-PCR was then used to analyze the presence of TR_s_ in hfPSMC cells from humans fetus (cell line) and in hAdultCC cells obtained from patients undergoing prosthesis implantation surgery (primary cultures). Total RNA obtained from hfPSMC and hAdultCC cells was amplified with the same oligomers used to study TR_s_ expression in rat tissues (Table 1) in fact, the oligomers were designed in receptor regions found to have almost 98% shared identity between human and rat. As observed in rats, all TR isoforms were found in hAdultCC cells, with the α2 isoform predominant, with an α2/α1 ratio of 1.28 ± 0.01 (Figure 5). Both α isoforms were present in hfPMSC cells, with greater α1 than α2 expression (α_2_/α_1_ ratio 0.87 ± 0.01). TR_β1_ expression increased between fetal and adult corpora cavernosa cells (Figure 5) (0.026 ± 0.021 vs. 0.71 ± 0.07 a.u.; *P* = 0.0027), reproducing the same ontogenetic profile as observed in rats.

## Discussion

In this study, we demonstrate for the first time the presence of TR_α1_, TR_α2_, and TR_β_ in rat and human corpora cavernosa. Although TR expression in animal [28–32] and human [9,27] male genital tissues is dramatically regulated by development, we were unable to demonstrate this in the penis. In fact, we found no difference in penile expression of TR_α1_ and TR_α2_ mRNA at any studied age. However, TR_β1_ mRNA did increase from the perinatal to adult age. The same expression pattern was observed with both RT-PCR and Northern blot experiments. The absence of an absolute quantification of TRs expression is a limitation of this study, but the complete parallelism between RT-PCR, Northern blot, and immunohistochemistry analysis gives consistency to our data.

In the primary cultures of cells obtained from rat corpora cavernosa of different ages, the expression of α1, α2 and β showed the same ontogenetic pattern, with similar levels of TR_α1_ and TR_α2_ in cells from both perinatal and prepubertal animals. This would seem to make TR_α_ regulation in the penis by steroid hormones very unlikely. In contrast, the β1 form is expressed at low levels in perinatal rats and increases in peripubertal rats, suggesting possible regulation by androgens; this is currently under exploration in our laboratories. However, the importance of TR_β1_ in the corpora cavernosa is unclear. In fact, while the TR_α1_ gene is widely expressed from early developmental stages, the TR_β_ gene is restricted until late embryogenesis, when it is induced in brain, pituitary and other tissues [33–35]. Furthermore, the β form is virtually absent in other male genital tissues, such as the testis [9]. On the other hand, while adult hyper- and hypothyroidism have some variable effects on gonadotropin secretion, testosterone transportation and testicular function [9], it is unlikely that the effect of thyroid diseases on sexual function can be solely due to androgen derangement.

The existence of two distinct genes encoding TR_s_ suggests that both are important for the T_3_-signaling pathway. However, the specific function of each form is not known, as they are often expressed with an overlapping pattern. TR knockout experiments have provided only a few hints of the function of a specific receptor, such as the importance of TR_α1_ on cardiac function or the development of the small intestine [8,10], or TR_β1_ in development of the ear [12]. Further studies are needed to explore the importance of TRs in the contraction of corpora cavernosa SMCs, which is essential for male sexual and reproductive function.

Finally, we analyzed the expression of TR_α1_, TR_α2_ and TR_β_ in human SMCs from adult and fetal penis. All three forms are present in the adult corpora cavernosa; however, although TR_α1_ and TR_α2_ are expressed at the same level as in adults in human fetal corpus cavernosum cells, the β form was almost absent. Although these data have been obtained in tissues characterized by vasculogenic impotence, we observed the same ontogenetic regulation in both rats and humans.

Several studies have underlined a correlation between thyroid function and male sexual function. In fact, a high prevalence of premature ejaculation has been found in hyperthyroid patients, whereas in hypothyroid subjects the main sexual complaint is delayed ejaculation [16,17,36]. While evidence of hypo- or hyperthyroidism is relatively rare in ED [15], we were the first to demonstrate that ED is a frequent symptom of male thyroid disease [17]. This result has been fully replicated by other researchers [18,19]. Both ejaculatory disorders and ED revert after achievement of euthyroidism. This suggests the direct involvement of thyroid hormones in the physiology of male sexual function.

Penile erection is largely due to the relaxation of SMCs within the corpora cavernosa. For this reason, we used immunohistochemistry to evaluate the cellular expression and localization of the TR prevalently expressed in the penis, the α1 isoform. Despite same limitations (data obtained only from 60-day-old animals), TR_α1_ has been demonstrated to be present in both endothelial cells and SMCs inside the corpora cavernosa and spongiosa, underlying its importance in SMCs contraction. The fact that we analyzed only TR_α1_ at protein level is another limitation of our study. However, the importance of the α isoforms is also confirmed by the fact that the α2/α1 ratio remains constant. In fact, it has been demonstrated that the alterations described in TR_α2_−/− mice are correlated with the modification of the α_2_/α_1_ ratio. Moreover, it seems that the tissue-specific difference in thyroid hormone responsiveness may depend on the relative amount of TR_α1_ expressed in each tissue [37].

The role of thyroid hormones in vascular function has been largely studied, but it is still not clear how thyroid hormones can impair relaxation of corpora cavernosa SMCs. In rabbits, hyperthyroidism impairs both the neurogenic and endothelium-dependent relaxation of *corpus cavernosum* smooth muscle. Alteration in SMCs function can be accompanied by adaptive changes in the smooth muscle contractile system. In a study on rabbits treated with thyroxine, Giuriato et al. [38] reported intimal thickening and up-regulation of non-muscle myosin in the aorta. It has been found that the thyroid state influences the density of α- and β-adrenoreceptors in smooth muscle, correlated with changed responses to catecholamines [39]. Also, hyperthyroidism leads to increased acetylcholine- and potassium chloride-induced contractions of urinary bladder strips [40] and increased acetylcholine-mediated relaxation of blood vessels [41]. Lofgren et al. [42] demonstrated that thyroid hormone treatment alters in vivo the isoform composition of myosin in fast and slow smooth muscles and that this change is sufficient to modify SMCs function. Studies of vascular smooth muscle (VSM) cells demonstrated the presence of iodothyronine deiodinase type II [43], suggesting that SMCs are a direct target for the actions of thyroid hormones. The identification of TR in both aortic and coronary VSM indicates the classic genomic action of T_3_ in these cells [44]. The effect of thyroid hormones on SMC also is also evident from studies of cardiac function. The direct effect of iodothyronines on cardiac myocytes, as well as its effects on peripheral vasculature, have also been demonstrated; in fact, the high systemic vascular resistance observed in patients with hypothyroidism is rapidly reversed with thyroid hormone treatment [45]. Moreover, studies using vascular smooth muscle cells isolated from rat aorta and cultured on a deformable matrix have demonstrated that exposure to T_3_ is able to relax these cells [44].

The demonstration of the presence of TRs in SMCs, together with the numerous observations of ED and/or ejaculatory disorders in thyroid conditions, support the hypothesis of a direct effect of thyroid hormones on *corpus cavernosum* SMCs. The presence of TR_s_ in these cells provides further evidence for this theory. In light of our results, the need for physicians to investigate the sexual function in men with thyroid disorders is increasingly important.
